# Reconstituting a two-step pathway for *N*,*N*-dimethyltryptamine (DMT) biosynthesis in bacteria

**DOI:** 10.1016/j.mec.2026.e00286

**Published:** 2026-07-21

**Authors:** Lucas Henrique Junges, Flavia Lada Degaut Pontes, Francisco José Teles Mota, Gustavo Passaglia Bruschi, Maria Paula Fernandes Bonaldi, Emanuel Maltempi de Souza, Marcelo Müller-Santos

**Affiliations:** a Department of Biochemistry and Molecular Biology, Nitrogen Fixation Laboratory, Federal University of Paraná (UFPR), Curitiba, Brazil; b Laboratory of Chromatography and Mass Spectrometry (LCEM), Department of Biochemistry and Molecular Biology, UFPR, Curitiba, Brazil

**Keywords:** DMT, Tryptamine, Tryptophan, Biocatalysis, Methyltransferase, *Corynebacterium*

## Abstract

N,N-dimethyltryptamine (DMT) is a bioactive indole alkaloid that could greatly benefit from scalable, fermentation-based production for research and pharmaceutical applications. In this study, we reconstructed a two-step bacterial pathway converting L-tryptophan to DMT via tryptamine. This involved combining a pyridoxal 5'-phosphate (PLP)-dependent tryptophan decarboxylase from the bacterium *Ruminococcus gnavus* (RgnTDC) with an *S*-adenosyl-L-methionine (SAM)-dependent *N*-methyltransferase from the cane toad *Rhinella marina* (RmNMT) in *Escherichia coli*. We optimised conditions for each step, determining 37 °C (pH 8.0) as the optimal condition for tryptamine production and 25 °C (pH 7.5) for DMT. While PLP supplementation did not raise tryptamine levels, methionine supplementation increased DMT levels by 2.8 times, emphasising the importance of methyl-donor supply. Co-culture and co-expression experiments showed that DMT accumulation depends on sufficient methylation capacity. Increased tryptophan availability led to tryptamine accumulation without a proportional increase in DMT formation, indicating a downstream limitation after decarboxylation. Together with the stimulatory effect of methionine supplementation, this result points to *N*-methylation and methyl-donor supply as key constraints in this system. In shake-flask cultures, a co-expression strain (TN1) produced 103 mg/L DMT after 48 h in complex medium without direct tryptophan supplementation. To enable growth in a defined medium, we used a workflow involving a tryptophan-enriched supernatant from a *Corynebacterium glutamicum* tryptophan overproducer, which supported de novo DMT formation at 16 mg/L in defined medium. These findings establish a plasmid-based platform for DMT production with *E. coli* and identify methyltransferase capacity as a key target for further yield improvements.

## Highlights

• Reconstituted a two-step DMT pathway (RgnTDC.→RmNMT) in *E. coli.*
• Whole-cell optima: 37 °C, pH 8.0 (tryptamine) and 25 °C, pH 7.5 (DMT).
• PLP did not increase tryptamine; methionine increased DMT titres by 2.8-fold.
• DMT plateaus despite tryptophan feeding, suggesting limited methylation capacity.
• *C. glutamicum* supernatant enables minimal-medium DMT production in a producer–converter workflow.

## 1 Introduction

*N*,*N*-dimethyltryptamine (DMT) is an indole alkaloid synthesised from L-tryptophan in both plants and animals (Barker, 2018). In the brain, DMT acts as a serotonergic agonist, primarily at 5-HT_2A_ receptors (Smith et al., 1998). Excessive serotonergic activation produces hallucinogenic or psychedelic effects, like other indole alkaloids, including psilocybin, bufotenine, and lysergic acid diethylamide (LSD) (Halpern, 2004). DMT is the main psychoactive ingredient in ayahuasca, a traditional brew prepared by Indigenous peoples of South America from native plants and used in ritualistic ceremonies (Simão et al., 2019). Interest in DMT has increased following evidence that psychedelic compounds may help treat psychiatric conditions such as post-traumatic stress disorder (Mitchell et al., 2023) and treatment-resistant depression (TRD) (Goodwin et al., 2022). DMT can also influence neuroplasticity (Calder and Hasler, 2023; Ly et al., 2018), motivating studies of its potential applications beyond mood disorders, including neurodegenerative conditions (Winkelman et al., 2023).

Developing pharmaceutical applications for DMT will require production methods that overcome the limitations of plant extraction. *Psychotria viridis*, one of the ayahuasca plants used as a source of DMT, can contain approximately 59 mg of DMT per gram of dry leaf (Cavalcante et al., 2018). However, this species has a long growth cycle, taking years to reach maturity, and it thrives mainly in mild-to-warm tropical climates, which limits cultivation regions worldwide. Additionally, DMT yields from *P. viridis* cultivated on farms can vary seasonally, influenced by factors like light, rainfall, and soil nutrients (Cavalcante et al., 2018). The lengthy process from planting to extraction would impact the market costs. Therefore, complementary platforms are needed to support controlled, scalable DMT production for research and pharmaceutical development.

Chemical routes for DMT synthesis have been described (Simoens et al., 2024), but microbial production offers a complementary strategy that operates under mild reaction conditions and can be integrated with metabolic engineering approaches (Abrahms et al., 2025; Sheldon and Woodley, 2018). Enzyme- and microorganism-based routes are also modular, allowing pathway tuning for DMT and, potentially, the generation of structural analogues. Because DMT biosynthesis requires a continuous supply of *S*-adenosyl-L-methionine (SAM) as the methyl donor for tryptamine methylation, genetically modified microorganisms have been investigated as production hosts (Abrahms et al., 2025; Junges and Müller-Santos, 2025; Sen and Jones, 2024).

Friedberg et al. constructed a synthetic DMT pathway in *E. coli* by expressing indolethylamine *N*-methyltransferase (INMT) from *Homo sapiens* (Axelrod, 1961; Thompson et al., 1999) and the tryptophan decarboxylase PsiD from *Psilocybe cubensis* (Blei et al., 2018; Fricke et al., 2017). Using genetic and process-optimisation strategies in benchtop fermenters, they achieved 74.7 ± 10.5 mg/L DMT with L-tryptophan supplementation under fed-batch conditions in a 2 L bioreactor. They also showed, for the first time, that *E. coli* can produce DMT de novo from glucose as the sole carbon source, reaching a maximum titre of 14.0 mg/L (Friedberg et al., 2023).

Chen et al. (2023) later expressed and characterised RmNMT, an indolethylamine N-methyltransferase from the cane toad *Rhinella marina*, in *E. coli*. In whole-cell biotransformation, *E. coli* expressing RmNMT converted 80 mg/L of tryptamine to 33 mg/L of DMT after 24 h (Chen et al., 2023). Kanis and colleagues characterised the substrate preferences of two enzymes involved in tryptamine-related biosynthesis: TrpM, a tryptophan *N*-methyltransferase from *Psilocybe serbica*, and PsiD, the tryptophan decarboxylase from *P*. *cubensis* in the psilocybin biosynthetic pathway (Kanis et al., 2024). They first generated mixtures of *N*-methylated tryptophans by chemical methylation of tryptophan, then semi-purified the products into fractions with different compositions (including fractions containing *N*,*N*-dimethyltryptophan, the direct precursor of DMT upon decarboxylation). They fed the methylated tryptophan fraction to *E. coli* expressing PsiD to monitor decarboxylation and DMT formation, achieving the highest DMT titre of 1.59 mg/L at 25 °C (Kanis et al., 2024). Although this titre is lower than those reported in other studies, the activity of PsiD towards *N*-methyltryptophan and *N*,*N*-dimethyltryptophan—yielding NMT and DMT, respectively—expands the known substrate scope of PsiD and points to an additional route for DMT biosynthesis in *E. coli*.

Beyond microbial hosts, Berman et al. recently reconstructed complete indolethylamine biosynthetic pathways in engineered plants, enabling the production of multiple psychedelic tryptamines in a single host (Berman et al., 2026). Notably, this work provided a proof of concept for the combinatorial expression of enzymes from different kingdoms to diversify tryptamine-derived alkaloids in plants. The authors also reported that DMT accumulation in the natural producer *P. viridis* peaks in young leaves, reaching up to 15.5 mg per gram of fresh tissue. By contrast, heterologous production of DMT in tobacco leaves was much lower, with a maximum of approximately 90 μg per gram of fresh biomass achieved using the plant-derived methyltransferase PvNMT1. Expression of the amphibian-derived RmNMT enzyme in tobacco resulted in almost no DMT accumulation. These observations suggest that although plant-based systems are powerful platforms for pathway reconstruction and molecular diversification, but their productivity may remain host- and enzyme-dependent. Moreover, plant-based production relies on biomass accumulation and remains sensitive to environmental and developmental variation, whereas microbial fermentation offers tighter process control, shorter production cycles, and more direct scalability.

Although the PsiD-human INMT pathway established the feasibility of microbial DMT production, the choice of pathway enzymes remains a key variable for improving catalytic efficiency and expanding the range of accessible tryptamine derivatives. Here, we investigated a DMT biosynthetic pathway that combines the bacterial tryptophan decarboxylase RgnTDC (McDonald et al., 2022) with the recently characterised *N*-methyltransferase RmNMT (Chen et al., 2023). RgnTDC is an attractive alternative to PsiD because it lacks a self-inhibition mechanism (Meng et al., 2025) and also exhibits an unusually broad substrate scope toward substituted tryptophan analogues (McDonald et al., 2022). RmNMT, in turn, catalyses the sequential *N*-methylation of tryptamine to DMT and accepts a broad range of substituted tryptamine analogues. Together, these features make the RgnTDC-RmNMT pair a promising modular platform for DMT biosynthesis and the production of structurally diverse dimethyltryptamine analogues.

After our experiments were completed, Abrahms et al. (2026) reported a genome-engineered *E. coli* platform based on the same RgnTDC–RmNMT enzyme pair. After promoter optimisation and chromosomal integration, this system reached 1.62 g/L DMT in a tryptophan- and glucose-fed bioreactor and 331 mg/L de novo DMT in a bioreactor without exogenous tryptophan, methionine or casamino acids (Abrahms et al., 2026). This work demonstrates the high potential of the RgnTDC-RmNMT pair in extensively engineered strains and provides a useful benchmark for plasmid-based configurations.

**Fig. 1:**
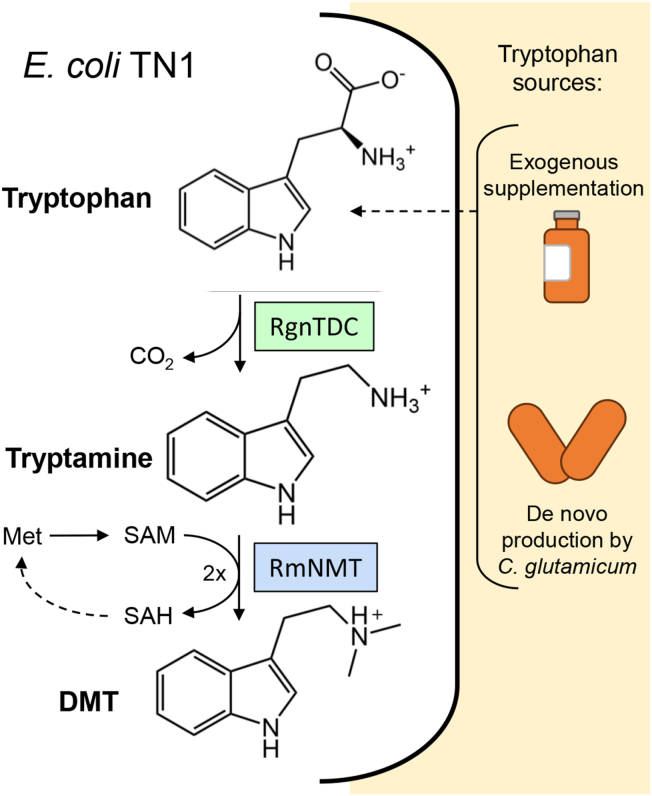
**Metabolic pathway for DMT production in *E. coli* TN1.** L-tryptophan is converted to tryptamine by the tryptophan decarboxylase RgnTDC from *Ruminococcus gnavus*, with release of CO_2_. Tryptamine is subsequently converted to *N,N*-dimethyltryptamine (DMT) through two sequential methylation reactions catalysed by RmNMT, an *N*-methyltransferase from *Rhinella marina*. Each methylation step uses *S*-adenosylmethionine (SAM) as the methyl donor and generates *S*-adenosylhomocysteine (SAH), which can be recycled through the methionine (Met) cycle. Tryptophan can be supplied either by exogenous supplementation or through de novo production by *Corynebacterium glutamicum* in the producer–converter workflow.

Here, we characterise a plasmid-based *E. coli* platform for DMT production from L-tryptophan by combining RgnTDC and RmNMT. We evaluate modular configurations, including co-culture, co-expression and a *C. glutamicum*/*E. coli* producer-converter workflow for defined-medium production. Although this study uses the same enzyme pair as Abrahms et al. (2026), it differs in that it implements co-expression across two plasmids, a configuration that may facilitate independent tuning of RgnTDC and RmNMT expression. We also identify potential bottlenecks that could be targeted in future work to increase DMT synthesis in *E. coli*. The pathway architecture and genetic configurations evaluated in this study are summarised in Fig. 1.

## 2 Methods

### 2.1 Bacteria, media, and growth conditions

Detailed information on the strains and plasmids used in the study is provided in Supplementary Table S1. *E. coli* strains were routinely cultivated in Lysogeny Broth (LB) at 37 °C and 180 rpm (Bertani, 1951). The *E. coli* Top10 strain was used for cloning procedures to propagate plasmids and maintain them as glycerol stocks. *E. coli* BL21(DE3) was used for enzyme overexpression for tryptamine and DMT production, as well as for the other experiments involving the conversion of L-tryptophan to DMT and the sequential co-culture workflow for de novo DMT synthesis. For tryptamine and DMT production, the defined medium Andrew's Magic Media (AMM) (Adams et al., 2022; He et al., 2015) and the complex medium Terrific Broth (TB) (Tartof and Hobbs, 1988) were used. The composition of AMM per litre contained: 3.5 g KH_2_PO_4_, 5 g K_2_HPO_4_, 3.5 g (NH_4_)_2_HPO_4_, 2 g casamino acids, 1 mL of 1 M MgSO_4_, 0.1 mL of 1 M CaCl_2_, 1 mL of thiamine HCl (0.5 g/L), 20 g glucose, and 100 mL of a 10× Mix solution. One litre of 10× Mix contained: 28 mg FeSO_4_·7H_2_O, 29.2 g NaCl, 5.1 g NH_4_Cl, 1.1 g MgCl_2_, 0.5 g K_2_SO_4_, and 0.2 mL of a micronutrient solution. One hundred millilitres of the micronutrient solution contained: 0.02 g (NH_4_)_6_Mo_7_O_24_, 0.12 g H_3_BO_3_, 0.01 g CuSO_4_, 0.08 g MnCl_2_, and 0.01 g ZnSO_4_. For AMM preparation, thiamine and micronutrient solutions were filter-sterilised through 0.22 μm polytetrafluoroethylene (PTFE) membranes. The phosphate-salt solution was prepared at twice the final concentration, adjusted to pH 7.0 or to the pH required for each experiment, and autoclaved separately. Glucose, salts, and other stock solutions were sterilised separately, as appropriate, and combined with the phosphate solution on the day of the experiment to avoid precipitation and medium darkening. The composition of TB per litre contained: 12 g tryptone, 24 g yeast extract, 8 mL of a 50% glycerol solution, 2.314 g KH_2_PO_4_, and 12.54 g K_2_HPO_4_. The potassium salts were adjusted to pH 7.5 and, together with glycerol, added to the remaining TB medium after autoclaving.

*C. glutamicum* TP679 carrying the vectors pCES208-trpEDEc and pECXT-Psyn (Kerbs et al., 2022), hereafter referred to as *C. glutamicum* Psyn, was routinely cultivated in Brain Heart Infusion (BHI) medium at 30 °C and 180 rpm. Alternatively, *C. glutamicum* Psyn was also cultivated in the minimal medium CGXII (minimal medium for *C*. *glutamicum*) (Keilhauer et al., 1993). The BHI medium contained per litre: 200 g calf brain infusion, 250 g calf heart infusion, 10 g peptone, 2 g dextrose, 5 g NaCl, and 2.5 g Na_2_HPO_4_ (final pH 7.4). The CGXII medium contained per litre: 20 g (NH_4_)_2_SO_4_, 5 g urea, 1 g KH_2_PO_4_, 1 g K_2_HPO_4_, 0.25 g MgSO_4_·7H_2_O, 42 g 3-(*N*-morpholino)propanesulfonic acid (MOPS), 10 mg CaCl_2_, 10 mg FeSO_4_·7H_2_O, 10 mg MnSO_4_·H_2_O, 1 mg ZnSO_4_·7H_2_O, 0.2 mg CuSO_4_, 0.02 mg NiCl_2_·6H_2_O, 0.2 mg biotin, and 40 g glucose. When required for the experiment, CGXII was supplemented with 0.25 g/L tyrosine and 0.25 g/L phenylalanine. For solid media, 15 g/L of bacteriological agar was added.

### 2.2 DNA manipulation

A codon-optimised gene for the expression of the *Ruminococcus gnavus* tryptophan decarboxylase (Swiss-Prot accession: A7B1V0.1; variant L355M (McDonald et al., 2022)) in *E. coli* was synthesised and cloned into the NdeI and XhoI sites of the pET-22b(+) by GenScript (Piscataway, NJ, USA). The sequence of an *N*-methyltransferase from *Rhinella marina* (GenBank accession: WKR38372.1) followed the yeast-codon-optimised design reported by Chen et al. (2023), which was originally intended to facilitate potential expression in *Saccharomyces cerevisiae* while reducing overabundant protein accumulation and inclusion body formation in *E. coli*. The gene was cloned into the NdeI and XhoI sites of pET-28a(+) by GenScript. The relevant information on coding sequences is provided in Supplementary Table S2. Other cloning procedures were performed using restriction endonucleases and T4 DNA ligase (Thermo Scientific) according to the manufacturer's recommendations. Correct assembly of recombinant plasmids was verified by diagnostic restriction digestion profiling, diagnostic PCR, and Sanger sequencing, following procedures previously published (Green and Sambrook, 2012).

### 2.3 Recombinant expression of enzymes related to tryptamine metabolism

The pET-22b_RgnTDC and pET-28a_RmNMT were individually transformed into CaCl_2_-competent *E. coli* BL21(DE3) by heat shock. For pre-inoculum, isolated colonies of these strains were picked with sterile loops and inoculated into 3 mL LB medium, cultivated for 14–16 h at 37 °C and 180 rpm, and then used to inoculate 5 mL of AMM medium in 50 mL penicillin glass vials for tryptamine and DMT production optimisation. Penicillin glass vials (Sigma-Aldrich #Z113999, 50 mL capacity; body diameter 43 mm, height 73 mm) served as small-volume screening vessels with a 5 mL working volume, enabling multiple parallel conditions to be incubated under identical shaking conditions. These vessels were used only for screening assays; larger baffled Erlenmeyer flasks were used for process-scale comparisons. For small-scale vial assays, tryptamine, tryptophan, indole and derivatives were dissolved in methanol. PLP and L-methionine were prepared in water and filter-sterilised before use.

#### 2.3.1 Optimisation of temperature and pH for tryptamine and DMT production by recombinant *E. coli*

*E. coli* BL21(DE3) strains transformed with plasmids carrying pET-22b_RgnTDC and pET-28a_RmNMT were pre-inoculated and cultivated as described above. A 1% (v/v) inoculum from the saturated culture was transferred into penicillin vials containing 5 mL of AMM medium at pH 7.0. Two hours after inoculation, 1 mM isopropyl β-D-1-thiogalactopyranoside (IPTG) was added to induce gene expression in both strains. In addition to the inducer, 500 μM L-tryptophan and 40 μM PLP were added to the strain expressing RgnTDC, whereas 500 μM tryptamine and 1.5 g/L (∼10 mM) L-methionine were added to the strain expressing RmNMT.

For the temperature test, cultures were moved to a different orbital shaker set to the target temperature and agitation speed. They were incubated for 10 min before the inducer, cofactors, and substrate were added. Cultures were incubated at different temperatures with shaking at 180 rpm for 4 h for *E. coli* expressing RgnTDC or 24 h for *E. coli* expressing RmNMT. The 4 h and 24 h incubation times used for the RgnTDC- and RmNMT-expressing strains, respectively, were selected to capture differences in early whole-cell conversion performance before substrate depletion or prolonged cultivation effects could mask condition-dependent differences. The substrate concentration was kept at 0.5 mM in these screening assays because L-tryptophan and tryptamine have limited aqueous solubility and were added from methanolic stock solutions; higher substrate concentrations would have required larger volumes of methanol, introducing an additional confounding variable. The initial pH was set by the phosphate buffer component before medium autoclavation. To adjust the pH of the AMM medium, HCl or KOH was added to the AMM buffer phosphate solution until the target pH was achieved. The pH-adjusted AMM buffer was then autoclaved. Cultures inoculated at various pH levels were incubated at the previously determined optimal temperature for 3 h for *E. coli* expressing RgnTDC or 18 h for *E. coli* expressing RmNMT.

#### 2.3.2 Effect of cofactors on tryptamine and DMT production by recombinant *E. coli*

To evaluate the effect of cofactor concentration on tryptamine and DMT synthesis, 2 h after inoculation of *E. coli* BL21(DE3) expressing RgnTDC or RmNMT, 1 mM IPTG and 500 μM of the respective substrate were added. In separate vials, a concentration gradient of pyridoxal phosphate (PLP) was applied for the RgnTDC-expressing strain, or a concentration gradient of L-methionine was applied for the RmNMT-expressing strain. Cultures were grown under the previously determined optimal production conditions: 37 °C and pH 8.0 for *E. coli* expressing RgnTDC, and 25 °C and pH 7.5 for *E. coli* expressing RmNMT. To determine production profiles, samples were collected at 2 and 4 h after induction for *E. coli* BL21(DE3) expressing RgnTDC, and a single sample was collected 24 h after induction for *E. coli* expressing RmNMT.

### 2.4 Production of tryptamine with resting *E. coli* cells

A 1% (v/v) inoculum from a saturated pre-inoculum of *E. coli* BL21(DE3) carrying the plasmid pET-22b_RgnTDC was transferred into 100 mL LB medium supplemented with ampicillin at 250 mg/L in a 500 mL Erlenmeyer flask and cultivated at 37 °C and 180 rpm. After 2 h, enzyme expression was induced with 1 mM IPTG. In addition, 40 μM PLP and 10 g/L glucose were added to favour carbon catabolite repression of the *E. coli* tna operon. The culture was incubated until OD_600_ > 4; cells were then centrifuged in 15 mL Falcon tubes, washed with 50 mM potassium phosphate buffer (pH 8.0), and resuspended to a final OD_600_ of 10 in 50 mM potassium phosphate buffer (pH 8.0) supplemented with 5 mM L-tryptophan, to a final volume of 5 mL.

One group received an additional 5 mM 2-deoxyglucose (2-DG) to test whether this glucose analogue could further reduce tryptophan degradation through catabolite-repression-related effects. Tubes were placed horizontally on an orbital shaker at 37 °C and 180 rpm. Every 24 h after tryptophan addition, cells were centrifuged (12,000 × g, 4 °C, 10 min), and the supernatant was collected and frozen for subsequent extraction and quantification of tryptamine. Cells were washed with one volume of cold 50 mM potassium phosphate buffer (pH 8.0), centrifuged at 4 °C, and resuspended in fresh 50 mM potassium phosphate buffer (pH 8.0) supplemented with 5 mM tryptophan and, for one group, 5 mM 2-DG. These substrate-renewal cycles were repeated for 6 days, after which the experiment was completed.

### 2.5 DMT production in *E. coli* co-cultures expressing RgnTDC and RmNMT

Two saturated pre-inocula of *E. coli* BL21(DE3) carrying either pET-22b_RgnTDC or pET-28a_RmNMT were grown under the appropriate antibiotic selection, centrifuged, and resuspended in fresh AMM medium to obtain an OD600 of ∼1.0 for both cultures. Different volumes of each culture were then used to prepare 1% (v/v) inocula in 5 mL of AMM medium in 50 mL penicillin vials. The RgnTDC: RmNMT ratios evaluated in the co-culture test were: 1:19, 2:18, 5:15, 10:10, 15:5, 18:2, and 19:1. After inoculation, vials were incubated at 37 °C and 180 rpm for 2 h, after which 1 mM IPTG was added, and cultures were supplemented with 500 μM L-tryptophan, 40 μM PLP, and 1 g/L methionine. The vials were then transferred to an orbital shaker and cultivated at 25 °C and 180 rpm for 24 h, after which the supernatants were collected for the extraction and quantification of tryptamine and DMT. For the co-culture experiments, no antibiotics were added to the AMM medium during the bioconversion stage.

### 2.6 Construction of *E. coli* strain TN1 for coexpression of RgnTDC and RmNMT

To construct an *E. coli* BL21(DE3) strain capable of producing DMT from L-tryptophan, the plasmids pET-22b_RgnTDC and pBBR1MCS-3 were digested with XbaI and XhoI, then ligated with T4 DNA ligase to generate pBBR1_RgnTDC. The pBBR1_RgnTDC and pET-28a_RmNMT were co-transformed into *E. coli* BL21(DE3), yielding the strain *E. coli* TN1.

The pBBR1MCS-3 backbone was selected because it is compatible with pET-28a_RmNMT in *E. coli* and allows the RgnTDC expression cassette to be transferred using the restriction–ligation workflow directly. Strain TN1 was cultivated in LB medium supplemented with tetracycline and kanamycin, as described above. Before construction of TN1, pBBR1_RgnTDC was transformed into *E. coli* BL21(DE3) and qualitatively checked for tryptamine production by TLC analysis of culture supernatants after IPTG induction and L-tryptophan supplementation.

#### 2.6.1 Effect of temperature on DMT production and enzyme solubility in *E. coli* TN1

Strain TN1 was cultivated in LB medium as described above. A 1% (v/v) inoculum from the saturated culture was transferred into penicillin vials containing 5 mL AMM medium (pH 7.5). Two hours after inoculation, 1 mM IPTG, 500 μM tryptophan, 40 μM PLP, and 1 g/L methionine were added. Cultures were grown at 25, 30 or 37 °C and 180 rpm, and supernatants were collected after 6 h and 24 h for product quantification. To evaluate the expression and solubility profiles of RgnTDC and RmNMT, *E. coli* TN1 was inoculated into 10 mL of AMM medium at pH 7.5. After 2 h of incubation, 1 mM IPTG, 500 μM tryptophan, 40 μM PLP, and 1 g/L methionine were added, and cultures were incubated at 25, 30, or 37 °C and 180 rpm for a further 6 h. Following induction, cells from 1.5 mL of culture were harvested by centrifugation. Pellets were resuspended in 500 μL of TS sonication buffer (50 mM Tris–HCl, pH 8.0; 150 mM NaCl; 20 mM imidazole). After sonication, the total protein concentration in each sample was determined using the Bradford assay (Bradford, 1976). Lysates were centrifuged at 20,000 × g for 10 min at 4 °C to separate soluble and insoluble fractions, and an amount corresponding to 5 μg of total protein from each lysate was analysed by denaturing polyacrylamide gel electrophoresis (SDS–PAGE) (Laemmli, 1970). Protein band densitometry was performed using ImageJ (Schindelin et al., 2012).

#### 2.6.2 Effect of substrate concentration on tryptamine and DMT production by *E. coli* TN1

Strain TN1 was cultivated in LB as described above. A 1% (v/v) inoculum from the saturated culture was transferred into penicillin vials containing 10 mL of Terrific Broth (TB) medium adjusted to pH 7.5. Two hours after inoculation, 1 mM IPTG, 40 μM PLP, 0.1 g/L methionine, and 0, 0.5, 1, 2, 5 and 10 mM L-tryptophan were added. Cultures were grown at 25 °C and 180 rpm, and samples were collected every 2 h up to 28 h and again at 48 h and 50 h to measure OD_600_ and collect supernatant for tryptamine and DMT analysis. Methionine was added from sterile-filtered aqueous stock solutions prepared at 30 g/L without pH adjustment. For tryptophan supplementation, the required amount was pre-aliquoted aseptically and added directly as a sterile solid to the flasks, where it dissolved progressively during shaking.

#### 2.6.3 Batch cultivation of *E. coli* TN1 for tryptamine and DMT production

From saturated cultures of *E. coli* TN1, 500 mL of AMM or TB medium containing 10 mg/L tetracycline and 50 mg/L kanamycin were inoculated into 2 L baffled Erlenmeyer flasks at 0.2% (v/v). Cultures were incubated at 37 °C and 180 rpm for 2 h. Four flasks were set up under different conditions to assess DMT production: (1) AMM (pH 7.5) without substrate supplementation; (2) AMM (pH 7.5) with 5 mM tryptophan supplementation every 24 h; (3) TB without substrate supplementation; and (4) TB with 5 mM tryptophan supplementation every 24 h. Two hours after inoculation, 0.005% Antifoam 204 (Sigma-Aldrich), 0.5 mM IPTG, and 40 μM PLP were added to all flasks. Flasks 1 and 2 were supplemented with 1 g/L methionine, whereas flasks 3 and 4 were supplemented with 0.1 g/L methionine. Flasks 2 and 4 received 5 mM tryptophan at induction (2 h post-inoculation) and again 24 h after induction (26 h post-inoculation). After induction, flasks were transferred to an orbital shaker at 25 °C and 180 rpm. Samples of 700 μL were collected every 2 h up to 28 h, and again at 48 h and 50 h, to measure OD_600_ and collect supernatants for tryptamine and DMT analysis. No samples were collected between 28 and 48 h. Cultures were incubated up to 50 h.

### 2.7 Sequential cultivation with *C. glutamicum* Psyn and *E. coli* TN1 for de novo DMT synthesis

The *C. glutamicum* Psyn strain was grown to saturation in BHI (Brain Heart Infusion) medium containing tetracycline (10 mg/L) and kanamycin (20 mg/L), then inoculated at 1% (v/v) into 50 mL of BHI or CGXII minimal medium supplemented with tyrosine and phenylalanine. Cultivation was carried out in 250 mL Erlenmeyer flasks at 30 °C and 180 rpm for 72 h to promote tryptophan accumulation in the medium. Cultures were then centrifuged, sterile-filtered through 0.22 μm PTFE membranes, and stored for subsequent use.

A saturated culture of *E. coli* TN1 was used to inoculate 50 mL of AMM or TB medium in 500 mL flasks at 1% (v/v). After 2 h at 37 °C and 180 rpm, 50 mL of filtered *C. glutamicum* BHI supernatant was added to *E. coli* TN1 grown in TB, and 50 mL of filtered *C. glutamicum* CGXII supernatant was added to *E. coli* TN1 grown in AMM. All conditions were induced with 0.5 mM IPTG and 40 μM PLP; the AMM/CGXII condition was supplemented with 1 g/L methionine, and the BHI/TB condition with 0.1 g/L methionine. Flasks were transferred to an orbital shaker at 25 °C and 180 rpm, and 700 μL samples were collected every 4 h to measure OD_600_ and evaluate product formation up to 52 h.

### 2.8 Extraction and quantification of tryptamine and DMT by HPLC

For tryptamine and DMT quantification, 500 μL of the culture supernatant to be analysed was collected, and 250 μL of 2 M NaOH and 750 μL of ethyl acetate were added to 1.5 mL microcentrifuge tubes. The tubes were vortexed at maximum speed for 10 s to promote phase mixing, and the upper organic phase was transferred to new tubes and dried under reduced pressure in a desiccator connected to a vacuum pump. The resulting residue was resuspended in 100 μL of methanol. Direct injection of culture supernatants was avoided to reduce particulate and medium-derived contamination of the HPLC system. Alkaline ethyl acetate extraction was therefore used as a sample-cleanup step for tryptamine and DMT. HPLC–UV analysis was performed with an Agilent 1220 Infinity LC equipped with a diode-array detector at 220 nm using a Kinetex C18 column (150 × 2.1 mm, 5 μm particle size; Phenomenex) under isocratic conditions, with a flow rate of 200 μL/min and a column oven temperature of 40 °C. The mobile phase consisted of water:methanol:formic acid (98:2:0.1, v/v/v), and the injection volume was 5 μL. For HPLC–UV quantification, tryptamine and DMT calibration curves (0-100 μM) were generated, using peak area and the resulting linear regression equation to calculate sample concentrations. Representative chromatograms, calibration parameters and extraction-recovery data are provided in Supplementary Figs. S1 and S2 and Supplementary Table S3. The efficiency of the liquid–liquid extraction procedure was evaluated by mixing sterile AMM medium with 0.5 mM tryptamine or DMT, followed by the same alkaline ethyl acetate extraction protocol used for culture samples. Mean extraction recoveries were 82.7 ± 5.6% for tryptamine and 88.7 ± 7.6% for DMT (n = 3). Therefore, the concentrations reported throughout this study represent conservative estimates of the actual metabolite concentrations present in the culture medium.

### 2.9 Statistical analysis

Most experiments were performed with at least three independent biological replicates, unless otherwise stated. Data are presented as mean ± standard deviation (SD), and individual replicate values are shown whenever possible. Statistical analyses were performed using GraphPad Prism version 11.0.0.

For experiments with a single factor, differences among groups were analysed using one-way analysis of variance (ANOVA), followed by Tukey's multiple-comparison test. For experiments involving two factors, such as cofactor concentration and sampling time, or temperature and enzyme/strain, data were analysed using two-way ANOVA with the interaction term, followed by Tukey's post hoc test when appropriate. When only two groups were compared, a two-tailed Student's *t*-test was used. Differences were considered statistically significant at *p* < 0.05. Statistical significance is indicated in the figures as follows: *p* < 0.05 (*)*, p* < 0.01 (**)*, p* < 0.001 (***), and *p* < 0.0001 (****).

## 3 Results

### 3.1 Benchmarking in vivo conditions for tryptamine and DMT production by engineered *E. coli*

The optimal in vivo catalytic conditions for the enzymes RgnTDC and RmNMT have not been previously evaluated. From an industrial perspective, determining the optimal in vivo conditions for these enzymes is particularly important, as supplying the necessary cofactors, such as PLP and SAM, would increase production costs. Nevertheless, these cofactors can be produced naturally by the bacterial host within an in vivo system.

To determine the ideal in vivo conditions for RgnTDC and RmNMT activities, *E. coli* BL21(DE3) cultures expressing the enzymes were cultivated in AMM (He et al., 2015).

AMM was used for the initial whole-cell optimisation assays because it is a rich semi-defined medium previously used in *E. coli* metabolic-engineering studies, including platforms for the production of psilocybin, norbaeocystin, DMT, and related tryptamine derivatives (Adams et al., 2019, 2022; Friedberg et al., 2023; Kanis et al., 2024).

**Fig. 2:**
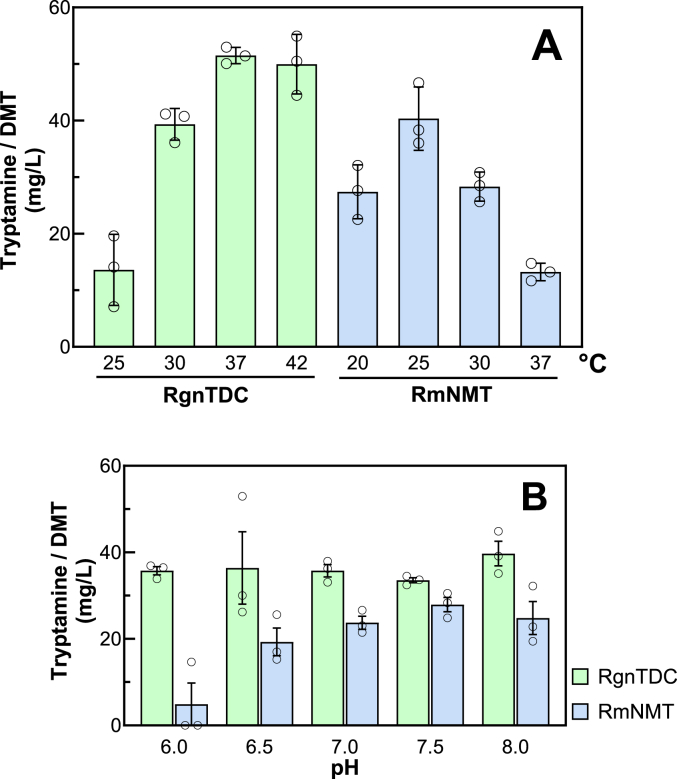
**In vivo optimisation of temperature and pH for tryptamine and DMT production in recombinant *E. coli*. (A)** Effect of incubation temperature on product formation by *E. coli* expressing RgnTDC (tryptamine; green) or RmNMT (DMT; blue). **(B)** Effect of initial medium pH on tryptamine (RgnTDC; green) and DMT (RmNMT; blue) production under otherwise constant conditions. Final culture pH after incubation was not measured in this experiment; therefore, the results reflect performance under the tested initial pH conditions. Bars represent mean ± SD; circles represent individual cultures.

We first examined how cultivation temperature (Fig. 2A) and medium pH (Fig. 2B) influence the production of tryptamine and DMT after induction and the addition of L-tryptophan for 4 h. When incubated at 37 °C, *E. coli* expressing RgnTDC produced significantly more tryptamine than cultures incubated at 25 and 30 °C, whereas no significant difference was observed between 37 and 42 °C (Fig. 2A). After 24 h of incubation, all L-tryptophan was depleted from the medium, yielding similar tryptamine levels across treatments (data not shown). For RmNMT, cultivation at 25 °C resulted in significantly higher DMT production than at 20 and 37 °C, whereas no significant difference was observed between 25 and 30 °C (Fig. 2A). Based on these results, 37 °C was selected for subsequent experiments with RgnTDC and 25 °C for experiments with RmNMT, as these temperatures yielded the highest titres.

We determined no statistically significant variation in tryptamine produced by RgnTDC-expressing *E. coli* across pH 6-8 (Tukey's test), with all tested cultures reaching ∼35 mg/L (Fig. 2B). However, at pH 8.0, the tryptamine produced was marginally higher compared to the other pH values (39.7 mg/L). The results of the cultivation at different pH levels using the RmNMT-expressing strain suggest that initial medium pH influences RmNMT-dependent DMT production under the tested conditions. The highest DMT production by *E. coli*-expressing RmNMT occurred at pH 7.5 (28 mg/L). A significant difference between groups was determined only at pH 6.0 and pH 7.5 (Tukey's test, p < 0.05), with the DMT produced at pH 6.0 being 5.7-fold lower than at pH 7.5 (Fig. 2B). Therefore, subsequent experiments were conducted at the optimal pH values: pH 8.0 for RgnTDC-expressing *E. coli* and pH 7.5 for RmNMT-expressing *E. coli*.

### 3.2 Cofactor supplementation assays: extracellular PLP provides no measurable benefit for RgnTDC, whereas methionine supports RmNMT-dependent DMT formation

**Fig. 3:**
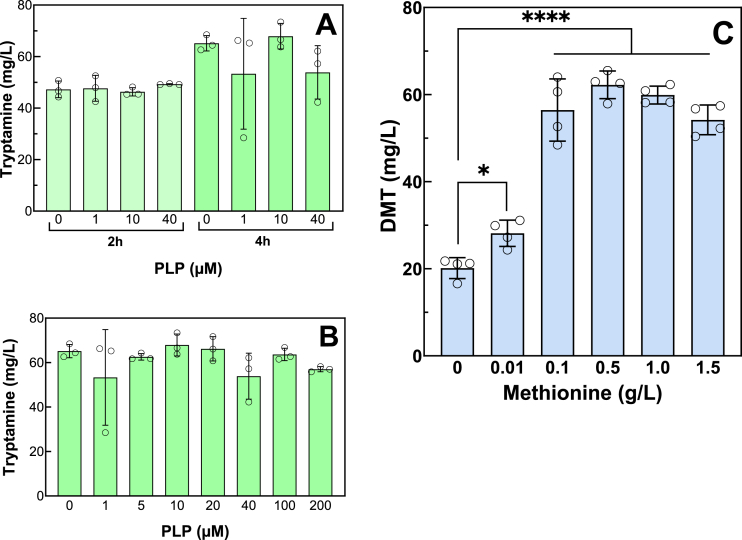
**Cofactor supplementation assays reveal no effect of PLP on tryptamine production, whereas methionine supports RmNMT-dependent DMT formation. (A)** Tryptamine titres at 2 h and 4 h after induction as a function of pyridoxal 5′-phosphate (PLP) concentration (0–40 μM). **(B)** Tryptamine titres across an extended PLP concentration range (0–200 μM). **(C)** Effect of L-methionine supplementation on DMT titres in *E. coli* expressing RmNMT. Bars represent mean ± SD; circles represent individual cultures. Statistical annotations: *p* < 0.05 (*)*, p* < 0.0001 (***) (one-way ANOVA with a post hoc multiple-comparison test, as indicated in Methods).

However, various PLP concentrations did not significantly increase in vivo tryptamine production (Fig. 3A and B). Only the group supplemented with 200 μM PLP exhibited significantly lower production compared to the group treated with 10 μM (Tukey's test, p < 0.05). Over-supplementing PLP in *E. coli* cultures can lead to unwanted side reactions involving the 4’-aldehyde group, disrupting amino acid metabolism and hindering cell growth (Ghatge et al., 2012; Sugimoto et al., 2017), which may account for the slight reduction of tryptamine at the highest [PLP] tested.

Groups supplemented with 0, 1, 10, and 40 μM PLP exhibited similar tryptamine levels at 2 and 4 h after substrate addition, indicating that extracellular PLP supplementation did not measurably improve tryptamine production under the tested conditions. These findings differ from previous in vitro studies, likely because *E. coli* has at least three PLP biosynthetic pathways that can fulfil its intracellular cofactor needs (Kim et al., 2010).

The enzyme RmNMT uses two SAM molecules to convert tryptamine into DMT, producing two SAH molecules. In *E. coli*, SAH is recycled through the methionine cycle, converting it to SRH, homocysteine, methionine, and eventually back to SAM. While direct SAM supplementation could boost cofactor levels, it is economically impractical for industrial use due to its high cost. Instead, methionine—the precursor to SAM—has lower added value and may enhance DMT production in *E. coli* expressing RmNMT. Previous research indicates that supplementing bacterial cultures with methionine increases the production of methyltransferase-catalysed products (Adams et al., 2019; Kunjapur et al., 2016). To assess how methionine concentration affects the culture medium, the RmNMT-expressing strain was cultivated in AMM supplemented with various methionine concentrations (Fig. 3C). A significant difference was detected between groups supplemented with 0.1–1.5 g/L methionine and the non-supplemented control group (Tukey's test, p < 0.0001). Notably, adding just 0.1 g/L methionine increased the medium DMT concentration by 2.8-fold, suggesting that methyl-donor supply contributes to the limitation.

**Fig. 4:**
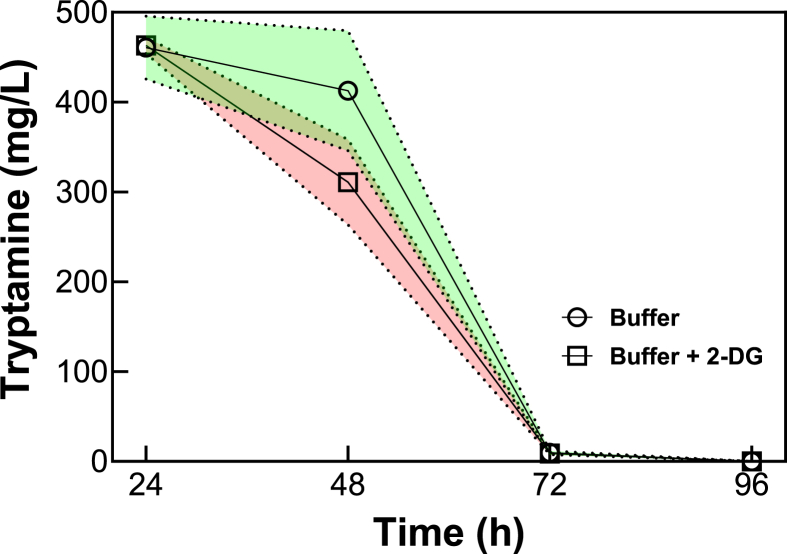
**Resting-cell conversion of L-tryptophan to tryptamine by RgnTDC-expressing *E. coli*.** Time course of extracellular tryptamine accumulation in a resting-cell assay performed in a buffer containing L-tryptophan, with or without 2-deoxyglucose (2-DG) as a catabolite-repressor agent. Lines show mean trends; shaded envelopes indicate variability across replicates (as shown in the plot).

The decarboxylation of tryptophan into tryptamine by RgnTDC is not part of any essential metabolic pathway in *E. coli*. Consequently, when the enzyme is expressed, cells can convert tryptophan into tryptamine independently of external carbon or nitrogen sources required for basal metabolism. To evaluate this production method, a resting-cell assay was performed using cells cultured in LB medium that expressed RgnTDC; these cells were subsequently resuspended in a buffer containing 5 mM tryptophan, with the supernatant collected and the buffer renewed every 24 h (Fig. 4).

Since endogenous *E. coli* tryptophanase TnaA can degrade tryptophan, producing pyruvate, indole, and ammonium, we investigated the use of catabolite repression to inhibit this competing pathway. Based on the reported regulation of the *tna* operon by carbon catabolite repression (Konan and Yanofsky, 1999), a high glucose concentration was included in the culture medium during RgnTDC expression and strain growth. We also investigated whether supplementation with 2-deoxyglucose (2-DG) could improve pathway performance through greater repression of *tna* operon expression. After 24 h of incubation, both treatments produced similar results, with residual tryptophan levels near 2.9 mM and a conversion rate of 58%. Indole was detected in both groups by thin-layer chromatography (TLC), indicating that tryptophan degradation occurred under both conditions, although it was not quantified. By 48 h, the group treated with 2-DG exhibited lower tryptamine levels than the untreated group. Therefore, under the conditions tested, supplementation with 2-DG did not improve pathway performance or reduce the apparent loss of tryptophan to competing metabolism.

### 3.3 Division of labour in *E. coli* co-culture enables DMT synthesis and identifies RmNMT as the rate-limiting step

**Fig. 5:**
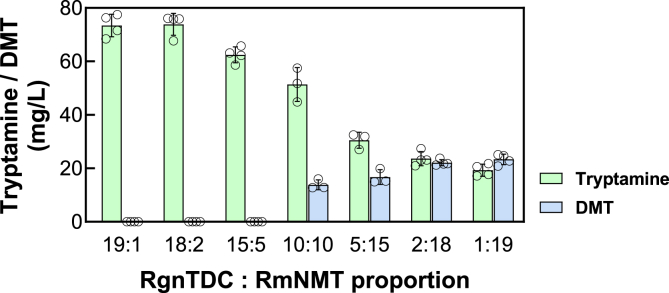
**Two-strain co-culture enables pathway modularisation and reveals ratio dependence of DMT accumulation.** Extracellular tryptamine (green) and DMT (blue) were produced in AMM by co-cultures containing different inoculation ratios of *E. coli* strains expressing RgnTDC or RmNMT. Bars represent mean ± SD; circles represent individual cultures.

Tryptamine and DMT are molecules that can cross cell membranes after production, resulting in their accumulation in the culture medium. To test whether pathway modularisation could support DMT synthesis, we designed a two-strain co-culture in which one *E. coli* strain expressed RgnTDC and the other expressed RmNMT (Fig. 5).

Different inoculation ratios of *E. coli* strains expressing RgnTDC and RmNMT were tested in AMM defined medium. Cultures were incubated at 25 °C and pH 7.5, conditions chosen because DMT production by RmNMT in vivo is slower than that of RgnTDC. Results showed that DMT could be synthesised from tryptophan using two different strains, each expressing one pathway enzyme. However, no DMT was detected when the RmNMT strain was inoculated at a lower ratio than the RgnTDC strain. The highest DMT concentration occurred at a 1:19 ratio (RgnTDC: RmNMT). To test whether the downstream bottleneck persists when both enzymes are expressed in the same host, we next constructed a dual-plasmid co-expression strain (TN1) and profiled the effects of cultivation temperature on DMT formation and RmNMT solubility.

### 3.4 Temperature profiling of the co-expression strain TN1 reveals RmNMT solubility as the primary bottleneck in DMT biosynthesis

**Fig. 6:**
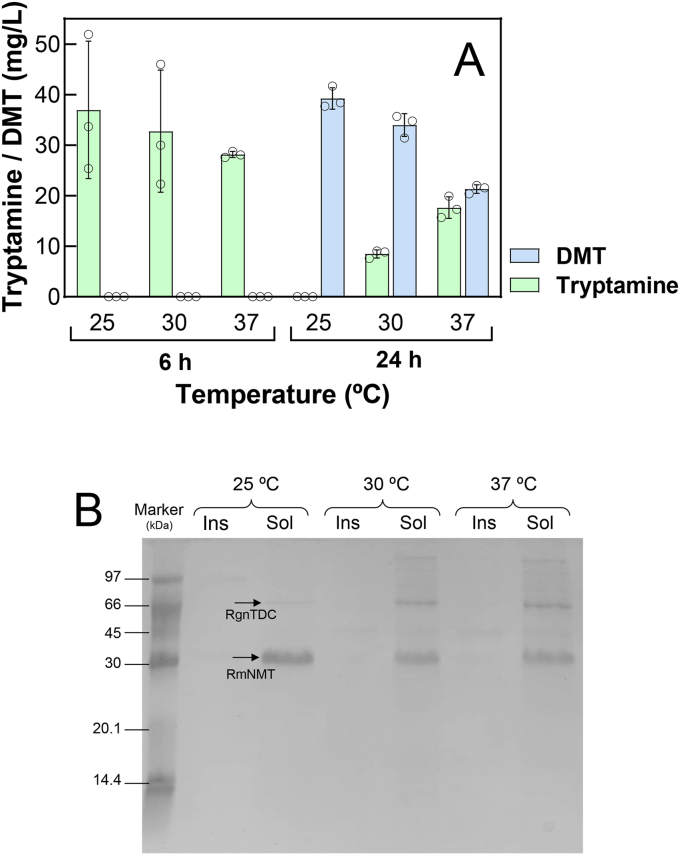
**Temperature-dependent DMT production and soluble expression profiles in the co-expression strain TN1. (A)** Extracellular tryptamine (green) and DMT (blue) produced by TN1 at the indicated temperatures after 6 h and 24 h post-induction. **(B)** SDS–PAGE analysis of insoluble (Ins) and soluble (Sol) fractions from TN1 cultures expressed at 25, 30, or 37 °C. Arrows indicate the bands corresponding to RgnTDC (60.2 kDa) and RmNMT (32.4 kDa).

The RgnTDC gene was inserted into the medium-copy-number vector pBBR1MCS-3 compatible with pET28a_RmNMT. The strain containing both plasmids was named TN1. Initial testing of TN1 focused on identifying the best temperature for DMT production from tryptophan (Fig. 6A). After 6 h of induction, no DMT was detected under any tested conditions. Although tryptamine levels at 37 °C were lower than at lower temperatures, the difference was not statistically significant (Tukey's test).

After 24 h of induction, only the culture at 25 °C showed no detectable tryptamine in the medium, and it achieved the highest DMT concentration among all treatments, reaching 208 μM (∼39 mg/L). DMT production at 25 °C was significantly higher than at 37 °C (Tukey's test, p < 0.01). Therefore, subsequent experiments with TN1 for DMT production were conducted at 25 °C.

To assess the solubility profiles of RgnTDC and RmNMT in TN1, an expression assay was conducted under conditions like those used in the temperature experiment, followed by protein gel electrophoresis (Fig. 6B). The culture at 25 °C exhibited the highest soluble RmNMT level, accounting for 7.4% of the total soluble protein, as determined by densitometric analysis (data not shown). This value represents lane-normalised band intensity rather than absolute protein abundance. Conversely, higher temperatures led to decreased soluble RmNMT levels, while RgnTDC levels increased. These findings support the idea that the reaction catalysed by RmNMT is the main rate-limiting step in DMT biosynthesis.

### 3.5 Tryptophan titration in TN1 reveals downstream methylation capacity as the main bottleneck

**Fig. 7:**
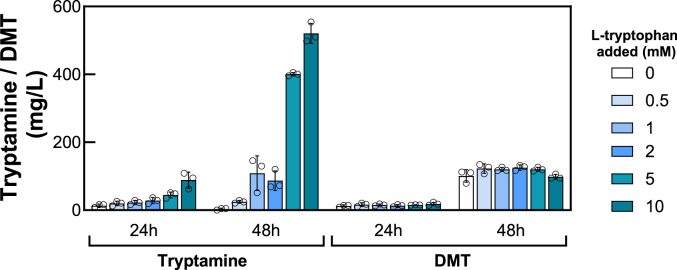
**Tryptophan titration uncouples the accumulation of intermediates from DMT formation in TN1.** Extracellular tryptamine and DMT titres in TN1 cultured in rich medium with increasing initial L-tryptophan concentrations (0–10 mM), quantified at 24 h and 48 h. Bars represent mean ± SD; circles represent individual cultures.

To identify the highest DMT concentration that strain TN1 could produce, we conducted a tryptophan dose-response experiment in complex medium (Fig. 7). After 24 and 48 h, the tryptamine levels in the supernatant increased proportionally with the amount of supplemented tryptophan, reaching up to 520.7 mg/L after 48 h with 10 mM tryptophan. Meanwhile, DMT levels remained relatively stable across different tryptophan supplementation levels, with a slight decrease at 0 and 10 mM (not statistically significant according to Tukey's test), yielding DMT levels of 100.5 and 98.3 mg/L, respectively. Although DMT levels were similar across treatments, the highest accumulation was observed with 2 mM tryptophan, producing 125.48 mg/L DMT. These results indicate that, in a TB medium, TN1 can sustain DMT formation without exogenous tryptophan supplementation, implying that tryptophan availability is not limiting under these conditions.

These results indicate that, in TB medium, TN1 can sustain DMT formation without exogenous tryptophan supplementation, implying that tryptophan availability is not the main limitation under these conditions. Instead, the accumulation of tryptamine without a proportional increase in DMT suggests a downstream limitation in the methylation step after tryptophan decarboxylation.

### 3.6 High-aeration flask cultivation of TN1 across media and feeding regimes reveals methylation constraints and growth–production trade-offs

To test whether process intensification could improve pathway throughput despite this downstream methylation constraint, we next evaluated TN1 in high-aeration flask cultivations across different media and tryptophan-feeding regimes. Experiments were carried out in 0.5 L of defined medium (AMM) or complex medium (TB), with and without tryptophan supplementation, over 50 h of cultivation.

**Fig. 8:**
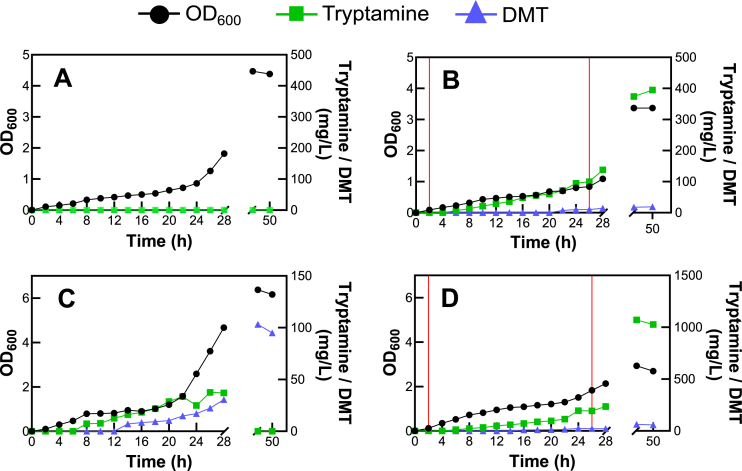
**High-aeration batch cultivation of TN1 in minimal and rich media highlights growth–production trade-offs.** Time-course profiles of OD_600_ (black), extracellular tryptamine (green), and DMT (blue) in baffled flasks under four conditions: **(A)** AMM without tryptophan supplementation; **(B)** AMM with L-tryptophan supplementation (red vertical lines denote addition time points); **(C)** TB without tryptophan supplementation; **(D)** TB with L-tryptophan supplementation (red vertical lines denote addition time points). Note differing y-axis scales across panels. Because no samples were collected between 28 h and 48 h, the 48-h value should be interpreted as the highest measured DMT concentration under this condition, rather than as the confirmed maximum titre of the cultivation. This experiment was performed as a single-process run per condition; therefore, these time-course profiles were interpreted descriptively and not subjected to statistical comparison.

Cultivation in AMM without supplementation reached a maximum OD_600_ at 48 h (Fig. 8A). Under these conditions, neither tryptamine nor DMT was detected at any sampled time point, indicating that TN1 does not produce sufficient endogenous tryptophan in AMM medium to sustain DMT accumulation. Supplementation with 0.51 g of tryptophan (5 mM) in AMM, performed at 2 and 26 h post-inoculation, resulted in a decrease in OD_600_ by the end of the fermentation period (Fig. 8B). Under the supplemented condition, tryptamine began to accumulate in the medium from 6 h onwards, reaching 396 mg/L after 50 h. Despite the high intermediate concentration, DMT production was detected only from 22 h onwards, reaching 18 mg/L at the end of the experiment.

When grown in a complex medium without supplementation, TN1 reached an OD_600_ of 6.37 after 48 h, roughly twice that of non-supplemented AMM (Fig. 8C). Under these conditions, tryptamine production began gradually from 8 h. However, after 50 h, it was no longer detectable in the medium. Under these minimally engineered, plasmid-based conditions, the culture accumulated 103 mg/L DMT after 48 h without direct tryptophan supplementation. This titre is higher than the de novo DMT titre reported by Friedberg et al. (2023) in *E. coli*, but lower than the titres recently achieved by Abrahms et al. (2026) using extensively engineered RgnTDC–RmNMT strains.

Despite the high-aeration conditions and increased cell density, the productivity in this culture was similar to that in the substrate titration experiment without supplements (Fig. 7), which showed tryptamine depletion and DMT production just over 0.5 mM after 48 h.

Tryptophan supplementation in TB medium resulted in a maximum OD_600_ of 2.93 after 48 h (Fig. 8D), indicating reduced cell growth. Under these conditions, the tryptamine concentration reached 1.26 g/L after 48 h, corresponding to a 66% conversion rate, which is higher than that observed in the substrate titration experiment with 10 mM tryptophan (32.5%; Fig. 7). These results indicate that cultivation conditions with higher aeration, which lead to higher cell density, favour the conversion of tryptophan to tryptamine.

The DMT concentration in the supplemented complex medium reached 63.3 mg/L after 48 h, which was lower than that observed under the non-supplemented condition. Because de novo production in minimal medium required an explicit tryptophan supply, we next implemented a producer–converter workflow using a tryptophan-overproducing *C. glutamicum* module to support TN1 under defined conditions.

### 3.7 A producer–converter workflow enables de novo DMT production in minimal medium via *C. glutamicum* tryptophan supply

Endogenous tryptophan production by TN1 in complex medium proved sufficient to sustain DMT synthesis, whereas in non-supplemented defined medium, neither tryptamine nor DMT was detected in the supernatant. However, DMT production in complex media such as TB introduces additional challenges for downstream purification and research use, primarily due to the medium's non-specific composition and the potential to co-purify undesired molecules (Nastouli et al., 2024). By contrast, production in a defined medium provides a reproducible cultivation composition and fewer downstream purification steps, though it requires exogenous tryptophan supplementation.

**Fig. 9:**
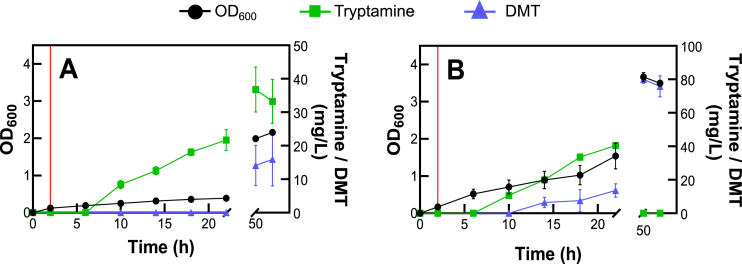
**The modular producer–converter workflow enables DMT formation under defined-medium operation.** Time-course profiles of OD_600_ (black), extracellular tryptamine (green), and DMT (blue) after addition of tryptophan-enriched, sterile-filtered *Corynebacterium glutamicum* Psyn supernatant (red vertical line) to TN1 cultures. **(A)** Minimal-medium production (AMM supplemented with CGXII-derived supernatant). **(B)** Complex-medium production (TB supplemented with BHI-derived supernatant). Error bars indicate variability as plotted.

With the aim of establishing a new strategy for de novo DMT production in a defined medium, we employed the bacterium *Corynebacterium glutamicum* TP679, which was engineered to overproduce L-tryptophan and carries the vectors pCES208-trpEDEc and pECXT_Psyn, referred to here as Psyn (Kerbs et al., 2022). Strain Psyn was cultivated in CGXII (minimal medium for *Corynebacterium glutamicum*) or BHI (Brain Heart Infusion) medium for 3 days to allow tryptophan accumulation in the supernatant. After this period, the supernatants were filtered and added to TN1 cultures 2 h after inoculation. Supernatant from CGXII cultivation was added to TN1 grown in AMM medium (Fig. 9A), whereas BHI-derived supernatant was added to TN1 grown in TB medium (Fig. 9B). DMT production was carried out in 500-mL Erlenmeyer flasks containing 100 mL of CGXII/AMM or BHI/TB medium for 52 h.

The AMM-medium culture reached an OD_600_ of 2.15 after 52 h, with a final DMT accumulation of 16 mg/L. In this culture, tryptamine accumulation was also observed after 52 h, indicating that the concentration of tryptophan produced by strain Psyn was sufficient to sustain DMT production in minimal medium.

The rich-medium culture reached an OD_600_ of 3.66 after 48 h, producing 79 mg/L DMT over the same period. No tryptamine accumulation was detected in the supernatant at the end of the experiment, indicating that the amount of tryptophan supplied by strain Psyn was not sufficient to saturate RgnTDC expressed by TN1 under these conditions.

## 4 Discussion

This study characterises a microbial platform for DMT production by reconstituting a two-step route from L-tryptophan via tryptamine in *E. coli* using the PLP-dependent decarboxylase RgnTDC and the SAM-dependent methyltransferase RmNMT (Fig. 2, Fig. 3). We first benchmarked in vivo operating conditions for each enzyme (Fig. 2) and then integrated the pathway into the co-expression strain TN1 to evaluate how cultivation regimes affect titre formation and intermediate pools (Fig. 6, Fig. 7, Fig. 8). Rather than simply demonstrating product formation, our data define a clear engineering diagnosis: pathway throughput is governed primarily by the methylation step, with RmNMT availability (in particular, soluble expression) and methyl-donor economy (SAM regeneration) emerging as the main optimisation targets (Fig. 3C, 6, 7-8). We further show that modular process architectures—such as step separation in co-culture and precursor-supply modules—can support defined-medium operation and improve process controllability (Fig. 5, Fig. 9).

### 4.1 Positioning relative to prior microbial DMT routes

Recent work has begun to explore enzyme-based and microbial routes to DMT, but the factors that determine titre plateau and operational robustness in vivo remain to be established (Chen et al., 2023; Friedberg et al., 2023; Kanis et al., 2024). In this context, our platform highlights enzyme choice as an engineering lever: RgnTDC enables rapid flux into tryptamine at enteric-like temperatures, while RmNMT provides a methylation module with broad substrate scope but strong temperature sensitivity in whole cells (Fig. 2A) (Chen et al., 2023; McDonald et al., 2022). Notably, DMT formation was favoured with methionine supplementation (Fig. 3C) and at lower temperature in the co-expression strain TN1 (Fig. 6A), and this condition coincided with the highest soluble fraction of RmNMT (Fig. 6B), supporting the *N*-methylation reaction as a principal determinant of whole-cell productivity in this system. This complements prior engineered *E. coli* routes and helps clarify why *N*-methylation can remain limiting even when upstream intermediates are plentiful (Friedberg et al., 2023).

### 4.2 Engineering diagnosis from titres and intermediate pools

A central feature of our dataset is that the concentration of the intermediate tryptamine responds strongly to alterations, whereas the final product titres often plateau, which is diagnostic of downstream capacity limitation. Increasing tryptophan availability in complex medium increased tryptamine accumulation but did not yield proportional increases in DMT (Fig. 7). A similar pattern emerged under process intensification: in high-aeration cultivation with tryptophan supplementation, tryptamine increased markedly, whereas DMT rose more slowly and to a lower final level than in the non-supplemented complex medium condition (Fig. 8C and D). This behaviour is consistent with a regime in which upstream decarboxylation supplies intermediates faster than downstream methylation can convert them, leading to intermediate accumulation rather than increased product formation. The co-culture experiment provided an independent line of evidence: DMT production depended strongly on the relative abundance of the RmNMT-expressing strain, and DMT was not detected when the methylation strain was underrepresented, consistent with a downstream step that must exceed a threshold capacity to draw flux through the intermediate pool (Fig. 5).

Although the co-culture experiment is consistent with the idea that methylation is the limiting step, co-culture systems present additional physiological constraints that can also reduce DMT formation. These constraints include the need for extracellular transfer of tryptamine between strains, potential diffusion limitations, competition for nutrients and cofactors, and dynamic changes in population composition during cultivation. Together, these observations converge on the conclusion that RmNMT-dependent methylation is rate-controlling under the tested conditions, while RgnTDC generally provides excess upstream tryptamine (Fig. 5, Fig. 6, Fig. 7, Fig. 8).

The resting-cell assay demonstrates that *E. coli* expressing RgnTDC can be reused for at least two 24-h tryptamine production cycles, with a loss of catalytic activity occurring after this period (Fig. 4). The decarboxylation step can be uncoupled from cell growth, potentially enabling high-cell-density biocatalytic processes in which substrate conversion is carried out by non-growing cells. Future studies should therefore consider using a strain with a *tnaA* gene deletion to prevent bacterial substrate degradation.

### 4.3 Cofactor economy explains why methylation, not decarboxylation, constrains throughput

The two enzymatic steps depend on distinct cofactor regimes, and our data indicate that these regimes impose qualitatively different constraints in vivo. Although RgnTDC is PLP-dependent, PLP supplementation did not increase tryptamine titres in whole cells across the tested ranges, suggesting that intracellular PLP supply is sufficient and that decarboxylation is not cofactor-limited in this context (Fig. 3A and B). At the highest PLP concentration tested, tryptamine production decreased (Fig. 3B). Excessive PLP has been reported to be detrimental to *E. coli* because it can trigger side reactions and perturb amino acid metabolism and growth (Ghatge et al., 2012; Sugimoto et al., 2017). By contrast, methylation imposes a direct and substantial demand on methyl-donor metabolism: two molecules of SAM are required per DMT, with concomitant formation of SAH, which must be recycled via the methionine cycle to sustain flux. In accordance with this, methionine supplementation substantially increased DMT accumulation (Fig. 3C), indicating that methyl-donor economy and SAM regeneration are limiting in vivo. This interpretation aligns with the broader literature on SAM-dependent methyltransferase biocatalysis, in which flux is often constrained by SAM availability and SAH accumulation under high methylation demand (Bennett et al., 2017; Kunjapur et al., 2016; Okano et al., 2020; Struck et al., 2012; Zhang et al., 2021).

### 4.4 Growth–production trade-offs and media dependence

The methylation bottleneck becomes particularly clear when interpreting growth and production jointly across cultivation regimes. In the defined medium without supplementation, TN1 reached moderate biomass but did not accumulate detectable tryptamine or DMT, indicating that tryptophan availability in this context is insufficient to drive measurable product formation (Fig. 8A). In TB medium without supplementation, TN1 accumulated DMT to the highest levels observed in this study (Fig. 8C), supporting the conclusion that complex medium provides sufficient tryptophan availability to sustain DMT formation without external feeding. However, this advantage comes with a process cost: undefined media complicate the extraction, quantification, and purification of small molecules and can increase the risk of co-extracting unwanted metabolites, a practical concern for producing research- or pharmaceutical-grade material.

Importantly, precursor feeding did not just raise titres directly. In AMM medium supplemented with tryptophan, tryptamine accumulated to high levels, whereas DMT appeared later and increased more gradually (Fig. 8B), consistent with methylation being limited by kinetic factors or physiological constraints at the culture level. In a complex medium, tryptophan supplementation markedly increased tryptamine but reduced both biomass and DMT titres relative to the non-supplemented rich-medium condition (Fig. 8D vs Fig. 8C). The reduced growth and DMT production under these conditions may be a consequence of known physiological sensitivities to indole-type compounds (Bennett et al., 2017; Chimerel et al., 2013; Struck et al., 2012).

### 4.5 Modular architecture for defined-medium operation and extensibility

To combine the control and reproducibility offered by defined media with de novo precursor supply, we used a modular producer–converter workflow. In this setup, tryptophan-rich supernatant from a *C. glutamicum* strain that overproduces tryptophan supported DMT formation by TN1 in defined medium (Fig. 9A) and enabled DMT formation in complex medium under the corresponding supernatant-transfer regime (Fig. 9B).

### 4.6 Outlook: strategies for improving DMT titre

Collectively, these results indicate that future improvements should prioritise increasing *N*-methylation reaction throughput by (i) improving soluble RmNMT availability and (ii) strengthening the methionine/SAM production and regeneration cycle (Fig. 3C, 6–8). Process strategies naturally follow from the observed temperature and solubility behaviour: a two-stage temperature regime could maximise biomass and enzyme accumulation during growth while favouring soluble RmNMT during production (Fig. 2A and 6). In addition, pH control and feeding strategies may stabilise physiology and methylation capacity, particularly because both steps operate within defined pH windows for optimal whole-cell performance (Fig. 2B), and feeding can exacerbate growth–production trade-offs (Fig. 8B–D). Finally, in situ product removal could help mitigate inhibition and toxicity associated with aromatic intermediates and products, potentially improving both growth and methylation during production.

## 5 Conclusions

In summary, we demonstrate a working microbial DMT platform and provide an engineering framework that explains why titres plateau under many conditions. The principal limitations lie downstream—through soluble RmNMT availability and SAM-dependent methylation capacity—rather than in tryptophan decarboxylation. Modular precursor supply using a *C. glutamicum* producer enables defined-medium operation and positions the platform for more controllable production.

Although the absolute DMT titres reported here are much lower than those recently achieved by Abrahms et al. (2026), it should be noted that their strains underwent extensive promoter optimisation, genome integration, and strain engineering. In contrast, the present study intentionally employed a minimally engineered platform to identify physiological bottlenecks governing DMT biosynthesis. Consequently, the engineering principles identified here may be directly applicable to further improve high-producing strains based on the RgnTDC-RmNMT pathway.

## Funding

Lucas Henrique Junges is a recipient of a studentship from the Postgraduate Programme of Biochemistry at the Federal University of Paraná, funded by the 10.13039/501100002322CAPES (Coordenação de Aperfeiçoamento de Pessoal de Nível Superior, 10.13039/501100002322Coordination for the Improvement of Higher Education Personnel), supported by the Ministry of Education in Brazil. Emanuel Maltempi de Souza and Marcelo Müller-Santos receive research grants from the 10.13039/501100003593CNPq (Conselho Nacional de Desenvolvimento Científico e Tecnológico; 10.13039/501100003593National Council for Scientific and Technological Development), with support from the Ministry of Science, Technology, and Innovation in Brazil.

## CRediT authorship contribution statement

**Lucas Henrique Junges:** Conceptualization, Data curation, Formal analysis, Investigation, Writing – original draft, Writing – review & editing. **Flavia Lada Degaut Pontes:** Data curation, Formal analysis, Investigation, Methodology, Writing – review & editing. **Francisco José Teles Mota:** Conceptualization, Data curation, Investigation. **Gustavo Passaglia Bruschi:** Conceptualization, Data curation, Investigation. **Maria Paula Fernandes Bonaldi:** Data curation, Investigation. **Emanuel Maltempi de Souza:** Conceptualization, Investigation, Writing – review & editing. **Marcelo Müller-Santos:** Conceptualization, Data curation, Formal analysis, Funding acquisition, Investigation, Project administration, Resources, Supervision, Writing – original draft, Writing – review & editing.

## Declaration of competing interest

The authors declare that they have no known competing financial interests or personal relationships that could have appeared to influence the work reported in this paper.

## Data Availability

Data will be made available on request.
