# Supplementary material for: Reconstituting a two-step pathway for N,N-dimethyltryptamine (DMT) biosynthesis in bacteria
Source: Metab Eng Commun. 2026 Jul 21;23:e00286. doi: 10.1016/j.mec.2026.e00286 (PMC13427508; doi:10.1016/j.mec.2026.e00286)
Supplement: Multimedia component 1 [file mmc1.docx]

**Supplementary Data**

**Reconstituting a two-step pathway for *N*,*N*-dimethyltryptamine (DMT) biosynthesis in bacteria**

Lucas Henrique Junges^1^, Flavia Lada Degaut Pontes^2^, Francisco José Teles Mota^1^, Gustavo Passaglia Bruschi^1^, Maria Paula Fernandes Bonaldi^1^, Emanuel Maltempi de Souza^1^, Marcelo Müller-Santos^1*^

This file includes Supplementary Method 1, Supplementary Figures 1 and 2, and Supplementary Tables 1-3

**Supplementary Method 1. Chromatogram processing and peak-area integration.** Chromatogram analysis was performed in the R environment (R Core Team, 2026) using the **tidyverse**, **ggplot2**, **dplyr**, **tidyr**, and **pracma** packages. First, to correct small instrumental variations in retention time between injections, chromatograms were aligned using the centroid of the chromatographic peak as a reference. The centroid was determined as the intensity-weighted mean retention time within the window corresponding to the peak of interest. The chromatogram of the highest-concentration standard was used as the reference for calculating the retention-time shift applied to the remaining chromatograms. This procedure reduces retention-time drift and improves comparability among analyses. After alignment, analyte quantification was performed by integrating the area under the chromatographic peak (AUC) within a window centred on the corresponding centroid. Peak areas were calculated using the trapezoidal rule implemented in the *trapz* function of the **pracma** package (Misra et al., 2019). The AUC values obtained for the dimethyltryptamine (DMT) and tryptamine (TRY) standards were used to construct analytical calibration curves via simple linear regression of integrated peak areas against analyte concentration. The regression equation and coefficient of determination (R²) were then estimated for each analyte. In addition, chromatograms from the analytical blank, tryptamine standard, DMT standard, and sample were plotted together as stacked chromatograms using the **ggplot2** package. To facilitate visual comparison and avoid signal overlap, a constant vertical offset of 1.0 × 10⁴ intensity units was applied between chromatograms while preserving peak shape and relative signal intensity. The display order was defined as follows: analytical blank, tryptamine standard, DMT standard, and sample.

**Supplementary Figure S1. Representative HPLC chromatograms for tryptamine and DMT analysis.**


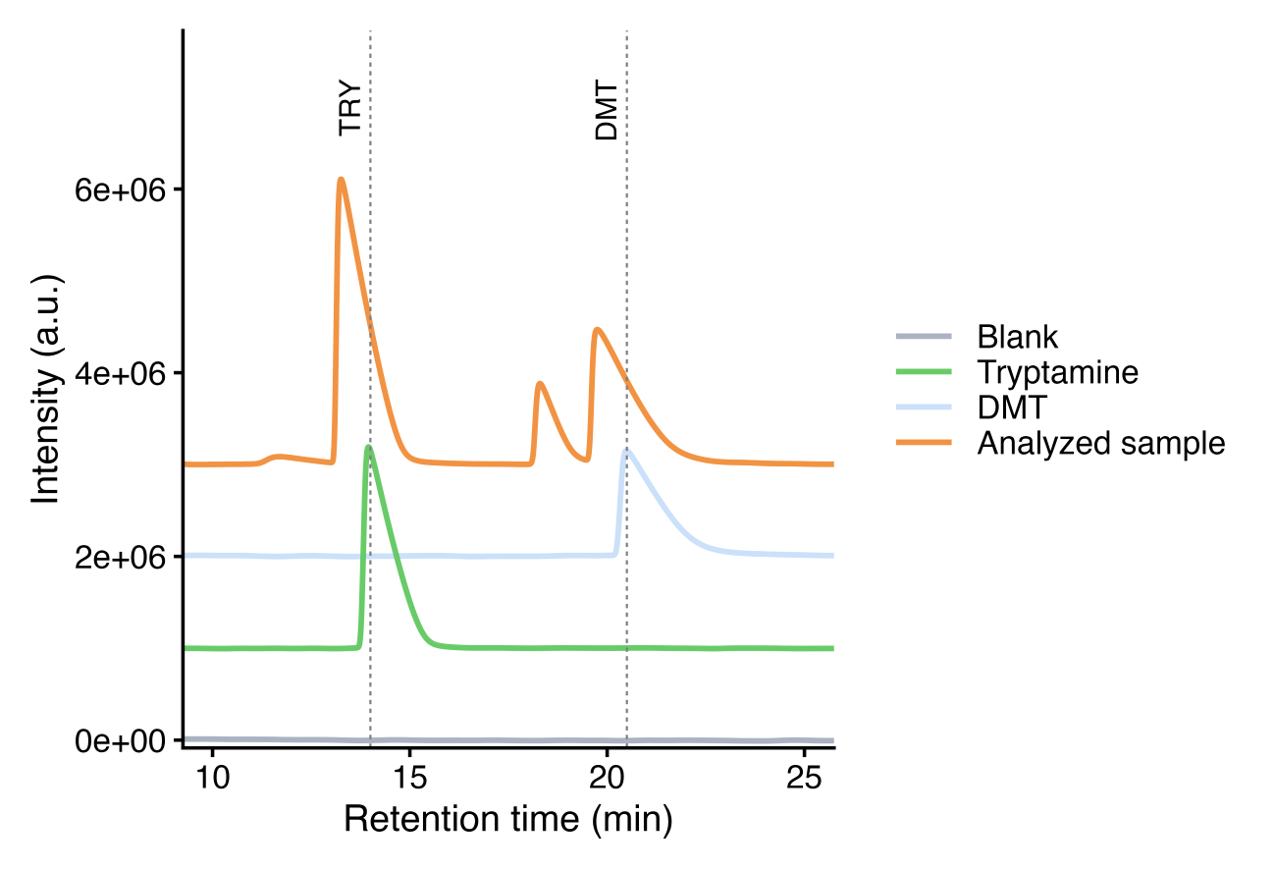


Representative HPLC–UV chromatograms showing separation of authentic tryptamine and DMT standards and representative culture extracts. Chromatograms demonstrate that DMT was resolved from earlier-eluting peaks under the chromatographic conditions used for quantification. DMT and tryptamine were quantified using calibration curves generated from authentic standards.

**Supplementary Figure S2. Calibration curves for quantification of tryptamine and DMT by HPLC–UV.**


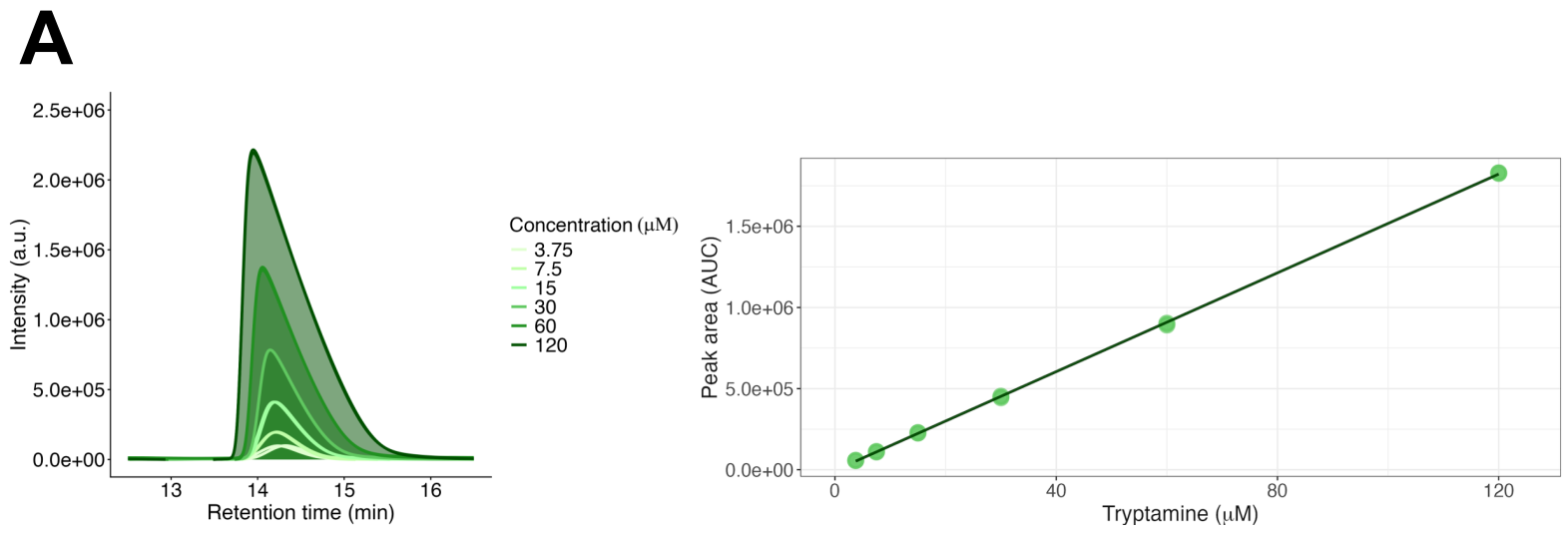


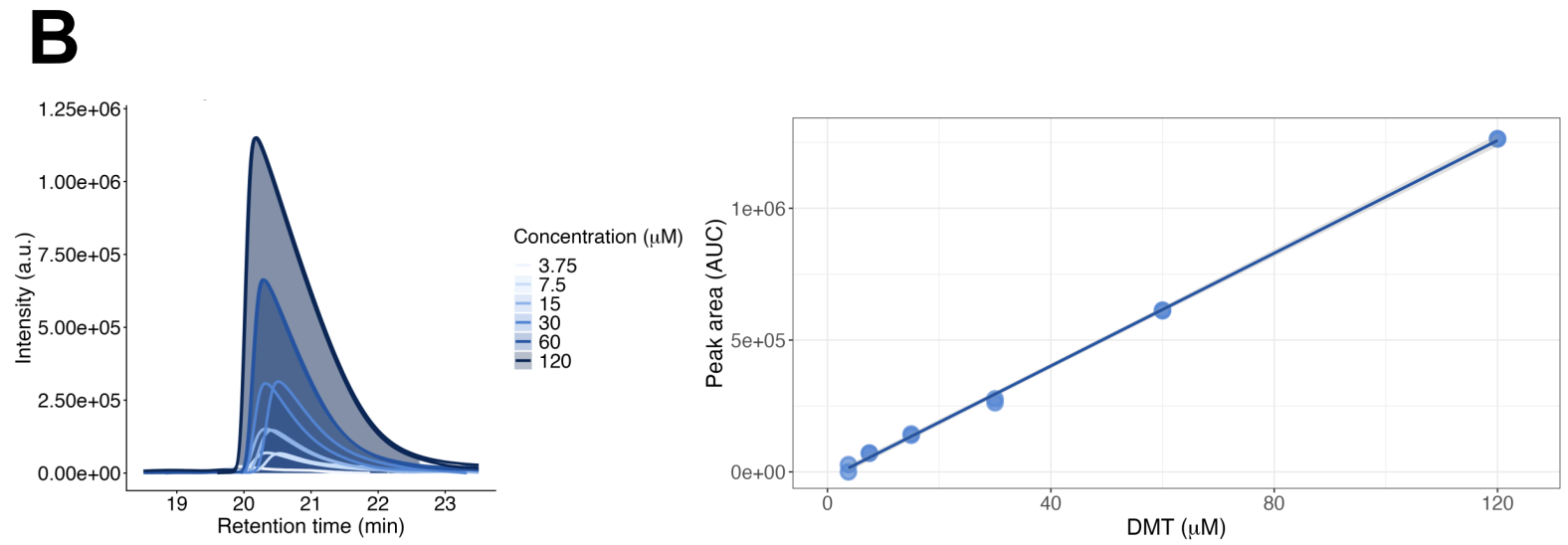


Calibration curves generated using authentic standards of tryptamine (**A**) and *N,N*-dimethyltryptamine (DMT) (**B**) under the chromatographic conditions used in this study. Peak area (AUC) was plotted against analyte concentration to quantify tryptamine and DMT in culture extracts. Regression parameters are provided in Supplementary Table S3.

**Supplementary Table S1. Strains and plasmids used in this study.**

| **Strain and plasmid** | **Description** | **Source/Reference** |
| --- | --- | --- |
| *Escherichia coli* | | |
| Top10 | *E. coli* K-12 derivative; genotype: F⁻ *mcrA* Δ(*mrr-hsdRMS-mcrBC*) φ80*lacZ*ΔM15 ΔlacX74 *recA1* *araD139* Δ(*ara-leu*)7697 *galU* *galK* *rpsL* (Str^R^) *endA1* *nupG*. Used for routine cloning, plasmid propagation and maintenance of recombinant plasmids. | Invitrogen/Thermo Fisher Scientific |
| BL21(DE3) | F⁻ *ompT* *hsdSB*(*rB⁻ mB⁻*) *gal* *dcm* λ(DE3); *E. coli* B derivative carrying the λDE3 prophage encoding T7 RNA polymerase under lacUV5 control; host for IPTG-inducible expression of RgnTDC and RmNMT. | (Studier and Moffatt, 1986) |
| TN1 | *E. coli* BL21(DE3) carrying the vectors pBBR1_RgnTDC and pET-28a_RmNMT | This work |
| *Corynebacterium glutamicum* | | |
| *C. glutamicum* Psyn | *C. glutamicum* TP679 carrying the vectors pCES208-trpEDEc and pECXT-Psyn | (Kerbs et al., 2022) |
| Plasmids | | |
| pBBR1MCS-3 | Broad-host-range, derived from pBBR1MCS; used here as the compatible vector backbone to construct pBBR1_RgnTDC for co-expression with pET-28a_RmNMT in *E. coli* TN1. | (Kovach et al., 1995) |
| pET-28a(+) | T7 promoter-based vector for N-terminal His-tag fusion; used here as the backbone for RmNMT expression in *E. coli* BL21(DE3) and TN1. | Novagen/Merck Millipore |
| pET-22b(+) | T7 promoter-based vector was used here as the backbone for RgnTDC expression in *E. coli* BL21(DE3). | Novagen/Merck Millipore |
| pET22b_RgnTDC | Vector for expression of RgnTDC through T7 promoter, Amp^R^ | This work |
| pBBR1_RgnTDC | Vector for expression of RgnTDC through T7 promoter, Tet^R^ | This work |
| pET28a_RmNMT | Vector for expression of RmNMT through T7 promoter, Kan^R^ | This work |

Bacterial strains and plasmids used for tryptamine and DMT production. The table includes genotype or relevant features, antibiotic resistance markers, experimental purpose, and source/reference. AmpR, ampicillin resistance; KanR, kanamycin resistance; TetR, tetracycline resistance.

**Supplementary Table S2. Synthetic coding sequences and expression cassettes used in this study.**

| RmNMT (*N*-methyltransferase from *Rhinella marina*; GenBank accession number: WKR38372.1)  Gene sequence  *catatg*gagaactcatactctgcacaaatgtacattgatgaatttgatcctgtacactactaccagacttactattcttcggggaaaggcggtatcgctagagaatggacagatttcgcattacagaatctgcatgaaaccttcggtccaggaggtgttaaaggagatatactaattgactttggcgctggacccacaatctatcaactgttgtctgcttgtgaagtgttcaacagtattataacctctgattttttagagcaaaacagggagcaattggaaaaatggttaagaaaggacccagatgccttagattggtctcactttactaagtatgtgtgtgaactggaaggtaatagagataactgggaaaaaaaggaagaaacgttgaggcgtaaagtcactaaagttttgaagtgtgatgctcttgcggaaaagccgtttgatgacgttcctatgccagaagccgactgtcttattagctgcttatgcttagaaaatccttgtcaagaccaagaagcctatatcaatatattgaaaaagcttaaggagctattgaaacccggtggtcacattataattcaaagtattcttaattgttcatactatcatataggtaacagctgcttctcccatctatccttatcgaaggatgacgtcgaaaaatcattcaaagaggcagggtatgaaattgttaaactaaaggtcttgccaagatccgtaatgtcagaaatggagatcagtgattctaatggctattattttatccatgctcgtaaaccacagaaagagtaa*ctcgag*  Protein sequence  MENSYSAQMYIDEFDPVHYYQTYYSSGKGGIAREWTDFALQNLHETFGPGGVKGDILIDFGAGPTIYQLLSACEVFNSIITSDFLEQNREQLEKWLRKDPDALDWSHFTKYVCELEGNRDNWEKKEETLRRKVTKVLKCDALAEKPFDDVPMPEADCLISCLCLENPCQDQEAYINILKKLKELLKPGGHIIIQSILNCSYYHIGNSCFSHLSLSKDDVEKSFKEAGYEIVKLKVLPRSVMSEMEISDSNGYYFIHARKPQKE |
| --- |
| RgnTDC (Tryptophan decarboxylase from *Ruminococcus gnavus* - variant L355M; Swiss-Prot accession: A7B1V0.1)  Gene sequence  *catatg*tcacaagtaataaagaaaaaaaggaatacgtttatgattgggactgagtatatcctgaactccacccagctggaagaagcgatcaagtccttcgttcacgacttctgcgcggaaaaacacgagattcacgaccagccggtggtcgttgaagcgaaagagcatcaagaggacaaaatcaaacagattaagatcccggagaagggtcgtccggttaacgaggtcgtgagcgaaatgatgaatgaagtttaccgctacagaggcgatgccaaccatccgcgttttttcagctttgttccgggtccggctagcagcgttagctggctcggtgacatcatgacctccgcgtataacattcatgcaggcggttcgaaactcgctccgatggtaaattgtattgagcaagaggtattgaagtggctggccaaacaggtgggcttcaccgagaacccgggtggtgttttcgtgagcggtggtagcatggcaaatattaccgcgctgacggccgcgcgtgataataaactgactgacattaacctgcatctgggtactgcgtacatcagcgatcagacccattcctccgtggccaaaggtctgcgcatcattggcattaccgactctcgtatcagacgtattccgaccaacagccactttcagatggataccaccaagttggaagaagcgattgagacggacaaaaagagcggctacattccgtttgtcgtcatcggcaccgcgggtacaaccaacaccggtagcatcgatccgctgaccgagatctcagcgttgtgcaaaaagcacgacatgtggttccacattgatggtgcgtacggcgcgagtgtgcttctgagcccgaagtataaatctctgctgacgggcaccggcctagctgatagcatttcttgggatgcacataagtggctgtttcagacctatgggtgcgcgatggtgttagttaaggacatccgcaatctgttccacagcttccatgttaacccggagtacctgaaagacttggagaatgatatcgacaacgtgaacacctgggatatcggcatggaaatgactcgtccagcgcgtggtttgaaattatggctgacgctgcaggttctcggctccgacctgatcggttcagcgatcgagcacggcttccaactggcagtttgggcagaggaagcgttgaacccgaagaaggattgggaaatcgtgtcgccagctcaaatggctatgattaactttcgttacgctcctaaggacctgacgaaagaggaacaagatatcctgaatgaaaagatctctcaccgcatcctggaaagcggttacgccgcaatctttaccacggtgttgaacggtaaaaccgttttgcgcatttgtgcgatccacccggaagctacccaagaagatatgcagcataccattgacttgcttgatcaatatggtcgtgaaatttataccgagatgaaaaaggcttaa*ctcgag*  Protein sequence  MSQVIKKKRNTFMIGTEYILNSTQLEEAIKSFVHDFCAEKHEIHDQPVVVEAKEHQEDKIKQIKIPEKGRPVNEVVSEMMNEVYRYRGDANHPRFFSFVPGPASSVSWLGDIMTSAYNIHAGGSKLAPMVNCIEQEVLKWLAKQVGFTENPGGVFVSGGSMANITALTAARDNKLTDINLHLGTAYISDQTHSSVAKGLRIIGITDSRIRRIPTNSHFQMDTTKLEEAIETDKKSGYIPFVVIGTAGTTNTGSIDPLTEISALCKKHDMWFHIDGAYGASVLLSPKYKSLLTGTGLADSISWDAHKWLFQTYGCAMVLVKDIRNLFHSFHVNPEYLKDLENDIDNVNTWDIGMEMTRPARGLKLWLTLQVLGSDLIGSAIEHGFQLAVWAEEALNPKKDWEIVSPAQMAMINFRYAPKDLTKEEQDILNEKISHRILESGYAAIFTTVLNGKTVLRICAIHPEATQEDMQHTIDLLDQYGREIYTEMKKA |

The table provides the synthetic DNA and deduced protein sequences of RmNMT, an N-methyltransferase from *Rhinella marina*, and RgnTDC, a tryptophan decarboxylase from *Ruminococcus gnavus*. Both coding sequences were codon-optimised *in silico* using the GenSmart tool from GenScript. The RmNMT sequence was optimised for expression in *Saccharomyces cerevisiae*, whereas the RgnTDC sequence was optimised for expression in *Escherichia coli*. Accession numbers, sequence features, cloning strategy, restriction sites, and vector context are included to document the constructs used for recombinant expression and to support reproducibility of the bacterial DMT biosynthesis system. The **NdeI** and **XhoI** restriction sites are highlighted in yellow and green, respectively, and the start and stop codons are underlined.

**Supplementary Table S3. HPLC–UV calibration and extraction recovery parameters.**

| Analyte | Calibration range (µM) | Average retention time  (minutes) | Regression  equation | Coefficient of determination (R²) | Extraction recovery  (±SD) |
| --- | --- | --- | --- | --- | --- |
| Tryptamine | 3.75–120 | 14.47 | y = 51.005x - 102.88 | 0.9901 | 82.7 ± 5.6% |
| DMT | 3.75–120 | 20.93 | y = 31.919x - 41.456 | 0.9993 | 88.7 ± 7.6% |

Analytical parameters for quantification of tryptamine and DMT by HPLC–UV after alkaline ethyl acetate extraction. The table includes retention time, calibration range, regression equation, coefficient of determination (R²), and extraction recovery from sterile AMM spiked with 0.5 mM analyte. Extraction recovery values are reported as mean ± SD from triplicate measurements.

**References**

Kerbs, A., Burgardt, A., Veldmann, K.H., Schäffer, T., Lee, J.H., Wendisch, V.F., 2022. Fermentative Production of Halogenated Tryptophan Derivatives with *Corynebacterium glutamicum* Overexpressing Tryptophanase or Decarboxylase Genes. ChemBioChem 23. https://doi.org/10.1002/cbic.202200007

Kovach, M.E., Elzer, P.H., Hill, D.S., Robertson, G.T., Farris, M.A., Roop 2nd, R.M., Peterson, K.M., 1995. Four new derivatives of the broad-host-range cloning vector pBBR1MCS, carrying different antibiotic-resistance cassettes. Gene 166, 175–176.

Misra, S., Wahab, M. F., Patel, D. C., & Armstrong, D. W. (2019). The utility of statistical moments in chromatography using trapezoidal and Simpson's rules of peak integration. *Journal of Separation Science*, 42, 1644–1657. <https://doi.org/10.1002/jssc.201801131>

R Core Team. (2026). *R: A language and environment for statistical computing*. R Foundation for Statistical Computing, Vienna, Austria. <https://www.R-project.org/>

Studier, F.W., Moffatt, B.A., 1986. Use of bacteriophage T7 RNA polymerase to direct selective high-level expression of cloned genes. J Mol Biol 189, 113–130.
